# Supplementary material for: Electrochemically induced crystalline-to-amorphization transformation in sodium samarium silicate solid electrolyte for long-lasting sodium metal batteries
Source: Nat Commun. 2023 Oct 16;14:6501. doi: 10.1038/s41467-023-42308-0 (PMC10579357; doi:10.1038/s41467-023-42308-0)
Supplement: Supplementary file 1 — Supplementary information [file 41467_2023_42308_MOESM1_ESM.pdf]

Supplementary Information

Electrochemically induced crystalline-to-amorphization transformation in sodium samarium silicate solid electrolyte for long-lasting quasi-solid-state sodium metal batteries

Ge Sun[1,†], Chenjie Lou[2,†], Boqian Yi[1], Wanqing Jia[1], Zhixuan Wei[1], Shiyu Yao[1]*, Ziheng Lu[3]*, Gang Chen[1], Zexiang Shen[1], Mingxue Tang[2]*, Fei Du[1]*

[1]Key Laboratory of Physics and Technology for Advanced Batteries (Ministry of Education), State Key Laboratory of Superhard Materials, College of Physics, Jilin University, Changchun, 130012, China.

[2]Center for High Pressure Science and Technology Advanced Research (HPSTAR), Beijing 100193, China.

[3]Department of Materials Science & Metallurgy, University of Cambridge, 27 Charles Babbage Road, Cambridge CB3 0FS, United Kingdom

[†]These authors contributed equally: Ge Sun, Chenjie Lou.

*Email: yaoshiyu@jlu.edu.cn (S. Y.); zluag@connect.ust.hk (Z. L.); mingxue.tang@hpstar.ac.cn (M. T.); dufei@jlu.edu.cn (F. D.)

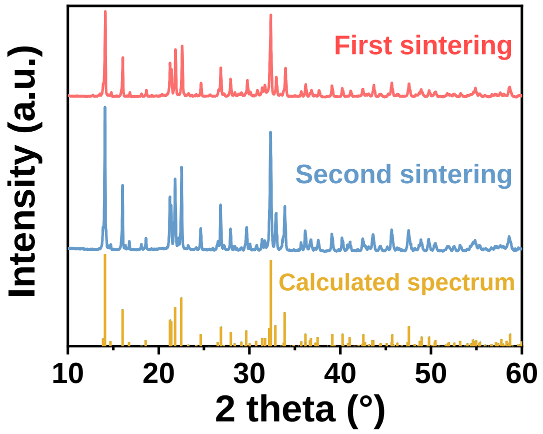

Supplementary Figure 1. XRD patterns at different sintering stages.

$Na_5SmSi_4O_{12}$ was successfully synthesized via a two-step solid-state reaction, first at 800 °C for 8 h and then 950 °C for 20 h. Pure $Na_5SmSi_4O_{12}$ can form after the first sintering. And the second sintering helps to achieve a dense ceramic pellet with few interfacial and bulk pores (Supplementary Fig. 2), beneficial to lower the grain boundary resistance and increase the ionic conductivity.

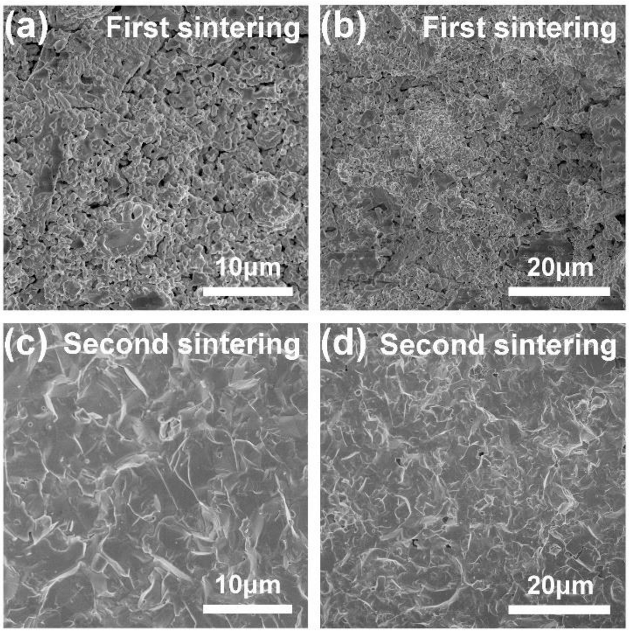

Supplementary Figure 2. SEM images at different sintering stages. (a) First sintering. Scale bar, 10 μm. (b) First sintering. Scale bar, 20 μm. (c) Second sintering. Scale bar, 10 μm. (d) Second sintering. Scale bar, 20 μm.

Pure $Na_5SmSi_4O_{12}$ can form after the first sintering (Supplementary Figure 1). And the second sintering helps to achieve a dense ceramic pellet with few interfacial and bulk pores, beneficial to lower the grain boundary resistance and increase the ionic conductivity.

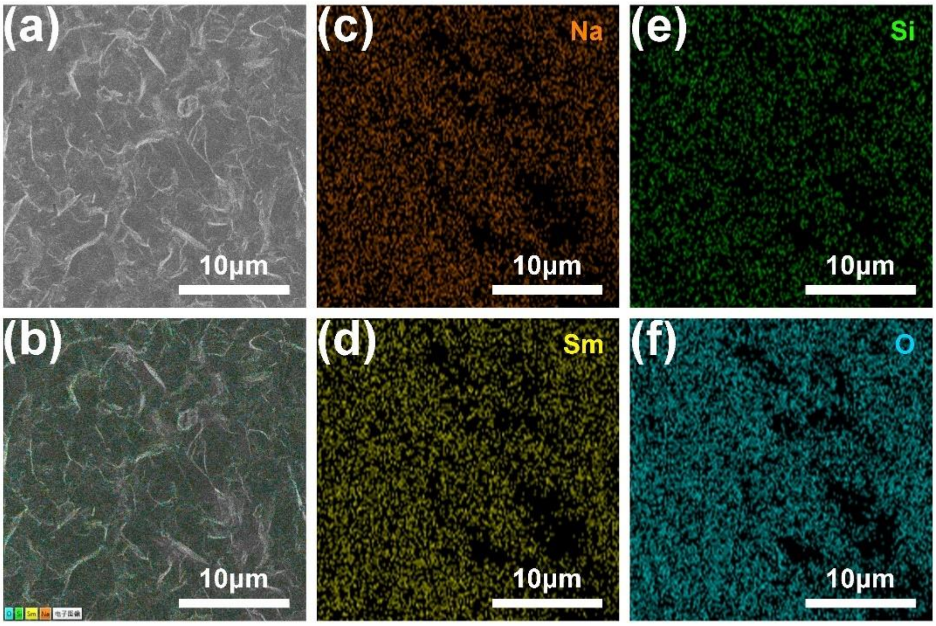

Supplementary Figure 3. The elemental mapping of the cross-section of the pelletized $Na_5SmSi_4O_{12}$. (a) SEM image. (b) Overlap elemental map of Na, Sm, Si, O. (c) Na elemental map. (d) Sm elemental map. (e) Si elemental map. (f) O elemental map.

The elemental mapping of the pelletized $Na_5SmSi_4O_{12}$ indicates that all the elements are uniformly dispersed in the monitored cross-section.

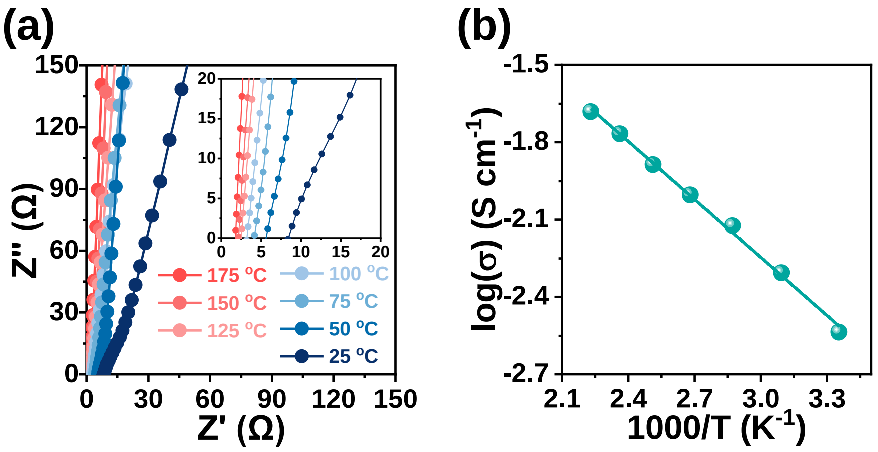

Supplementary Figure 4. (a) Nyquist plots of $Na_5SmSi_4O_{12}$ from 25 to 175 °C. (b) Arrhenius plot of the conductivity values for $Na_5SmSi_4O_{12}$.

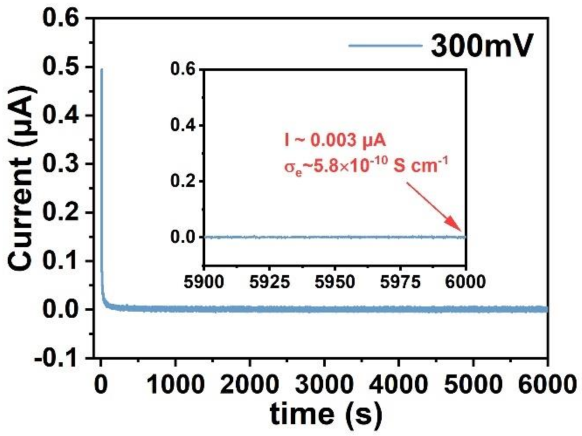

Supplementary Figure 5. DC polarization curve for $Na_5SmSi_4O_{12}$ under the applied voltage of 300 mV at room temperature. The inset shows the enlarged curve ranging from 5900 to 6000 s.

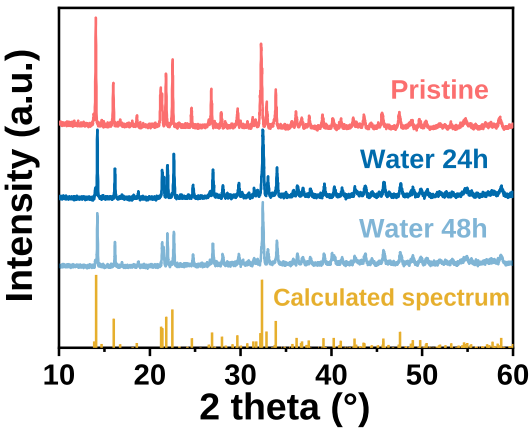

Supplementary Figure 6. XRD patterns after soaking in deionized water for 24 and 48 h.

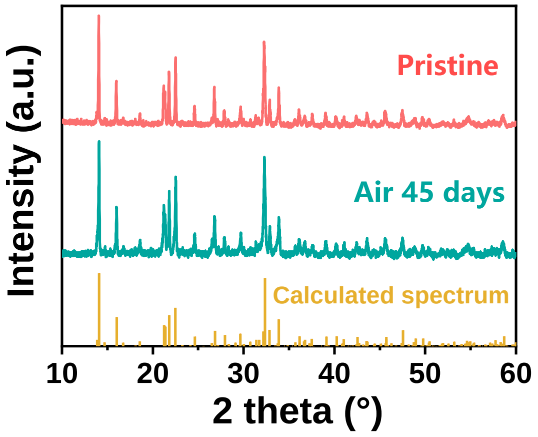

Supplementary Figure 7. XRD patterns after exposing to air for 45 days.

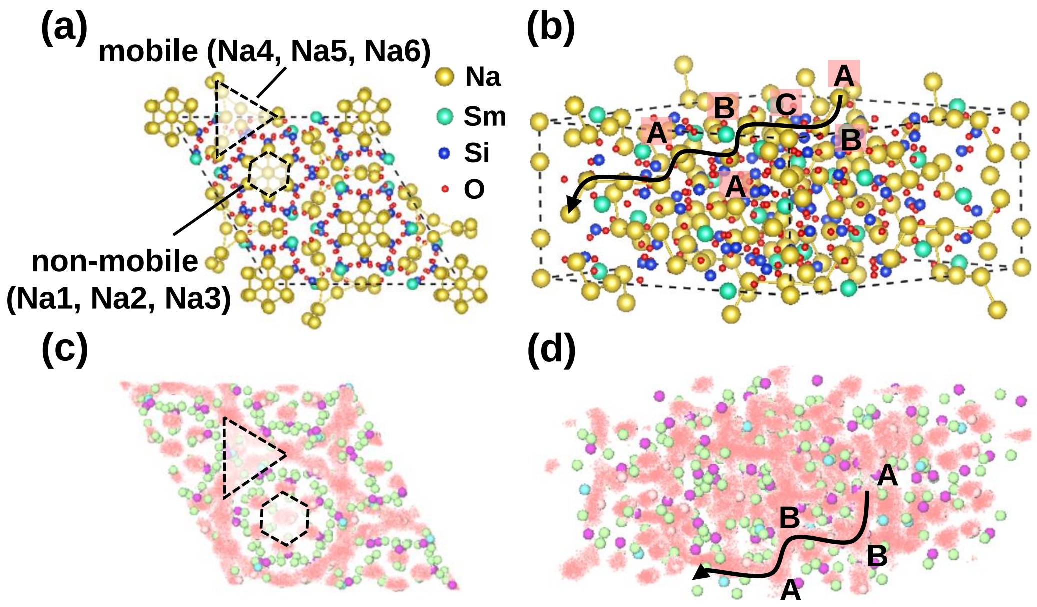

Supplementary Figure 8. (a) Top-down and (b) perspective view of crystal structures of crystalline $Na_5SmSi_4O_{12}$. (c, d) Molecular dynamics simulation trajectories of $Na_5SmSi_4O_{12}$.

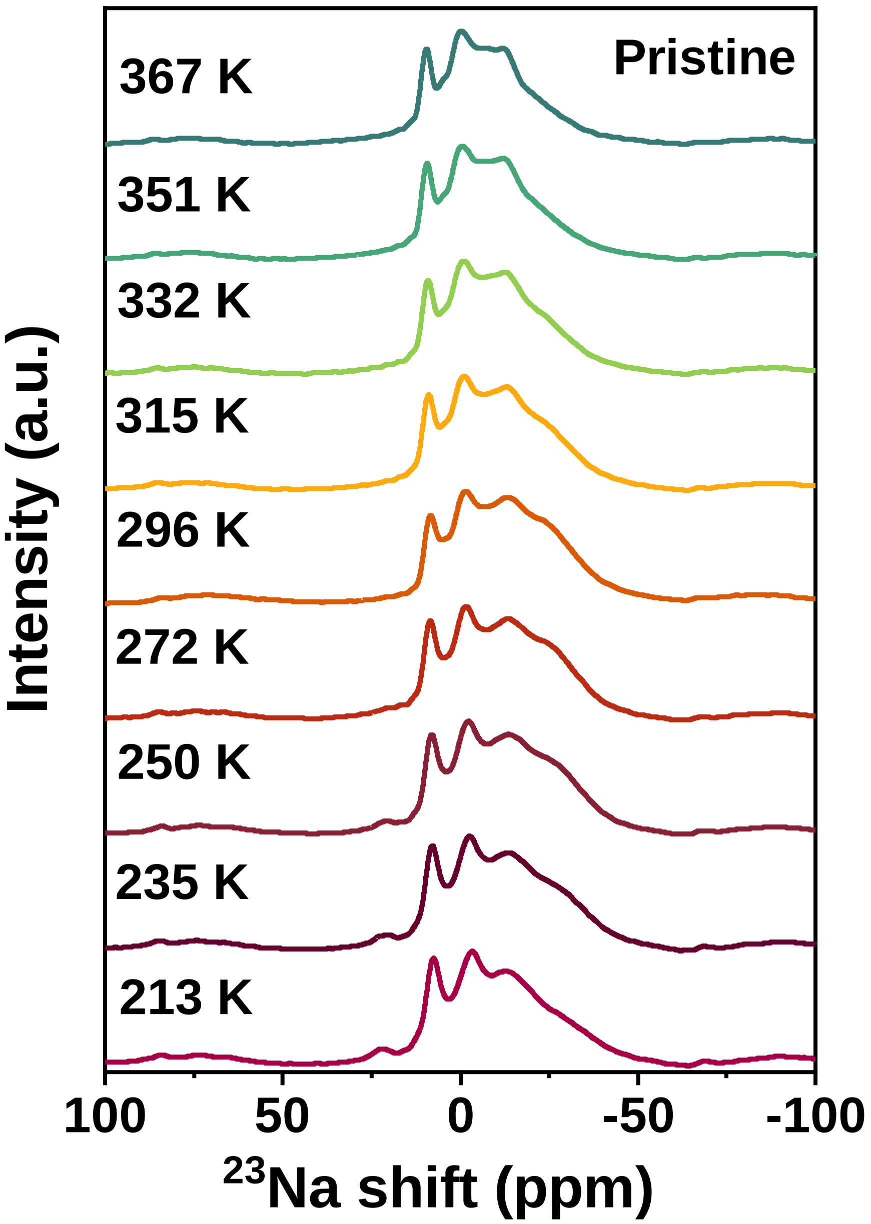

Supplementary Figure 9. Solid-state ^{23}Na NMR spectra of pristine crystalline $Na_5SmSi_4O_{12}$ at different temperatures.

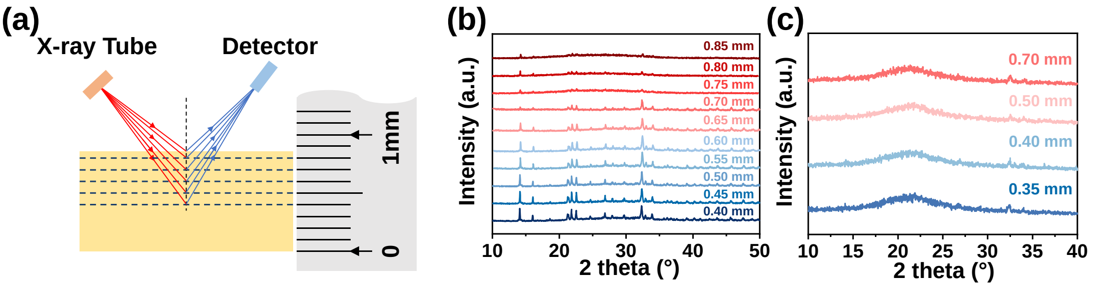

Supplementary Figure 10. (a) Schematic and XRD patterns of the $Na_5SmSi_4O_{12}$ polished to different depths after cycling (b) 100 h and (c) 800 h at 0.15 mA cm^{-2}.

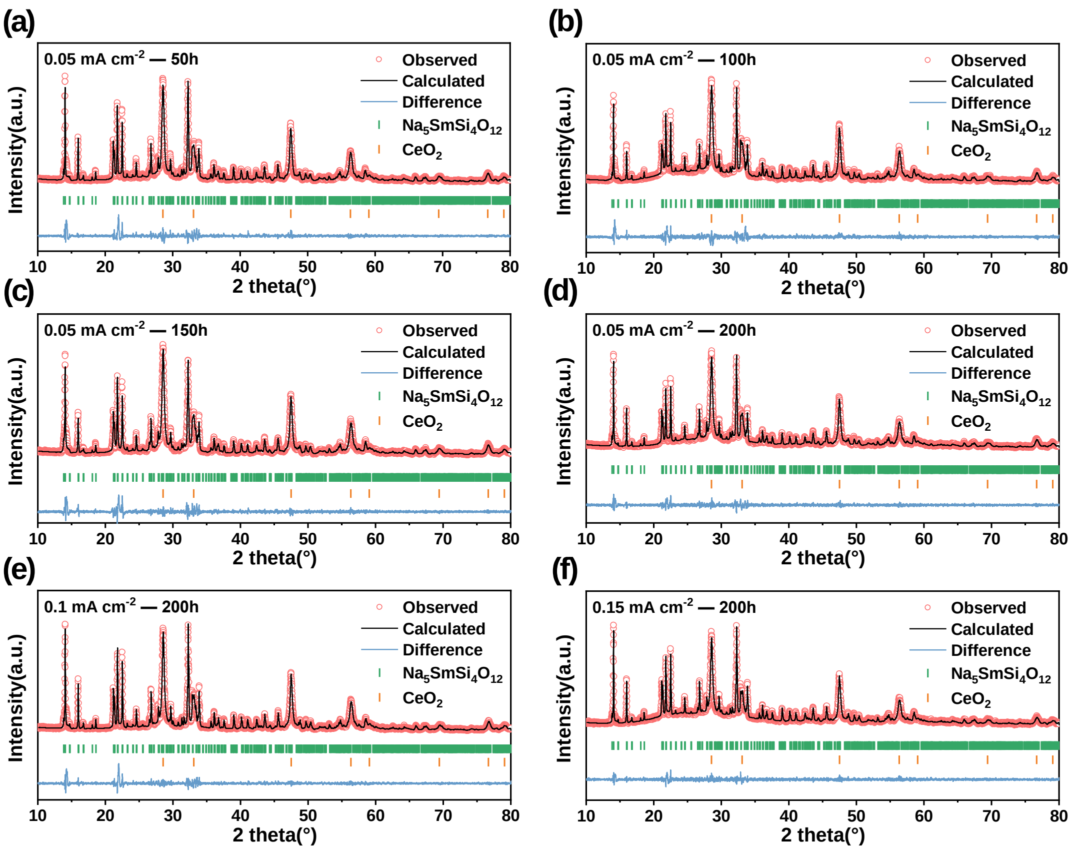

Supplementary Figure 11. Rietveld refinement based on the internal standard method by mixing CeO_2 with the $Na_5SmSi_4O_{12}$ at different plating/stripping stages. (a) At 0.05 mA cm^{-2} for 50 h. (b) At 0.05 mA cm^{-2} for 100 h. (c) At 0.05 mA cm^{-2} for 150 h. (d) At 0.05 mA cm^{-2} for 200 h. (e) At 0.1 mA cm^{-2} for 200 h. (f) At 0.15 mA cm^{-2} for 200 h. The corresponding Rietveld refined crystallographic data were summarized in Supplementary Table 5.

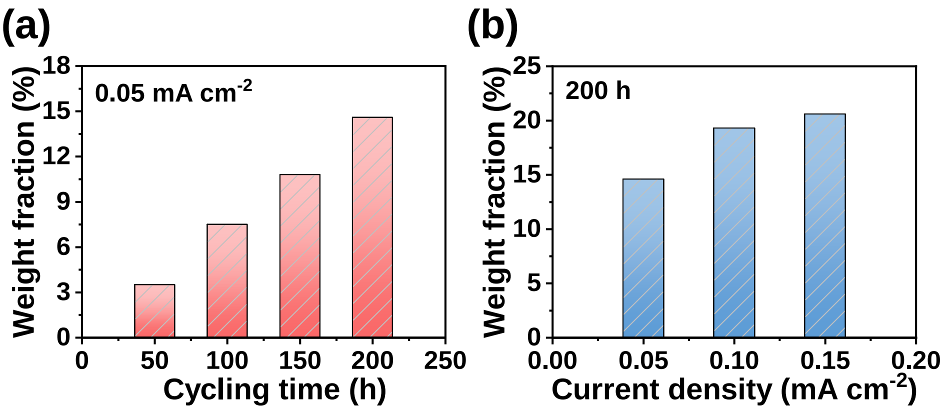

Supplementary Figure 12. The relationship between amorphization weight fraction and (a) cycling time and (b) current density.

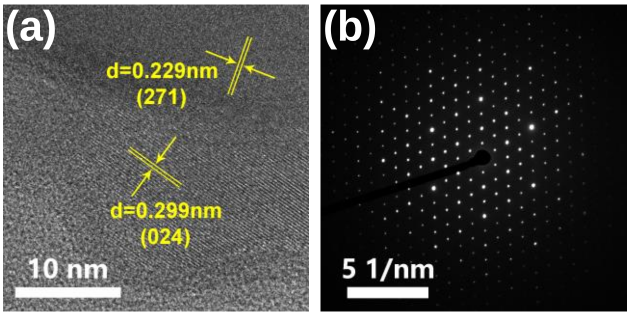

Supplementary Figure 13. (a) HRTEM and (b) SAED patterns of crystalline $Na_5SmSi_4O_{12}$.

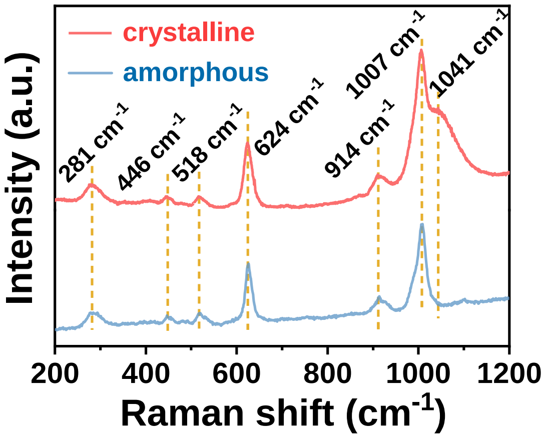

Supplementary Figure 14. Raman spectrum of $Na_5SmSi_4O_{12}$ before and after cycling.

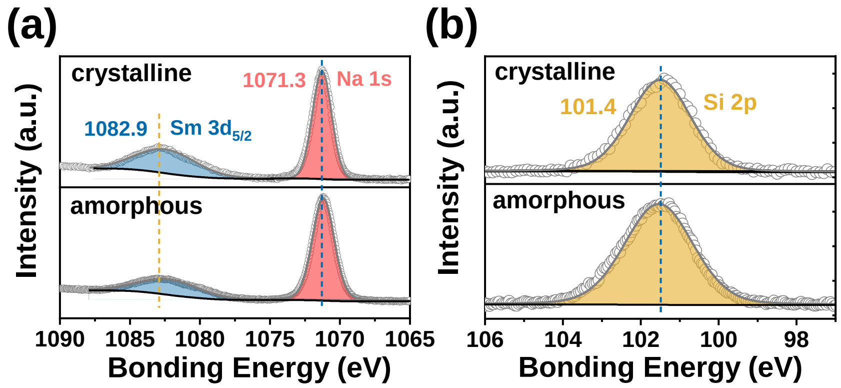

Supplementary Figure 15. (a) Sm 3*d*, Na 1*s* and (b) Si 2*p* XPS spectra of $Na_5SmSi_4O_{12}$ electrolyte at different states.

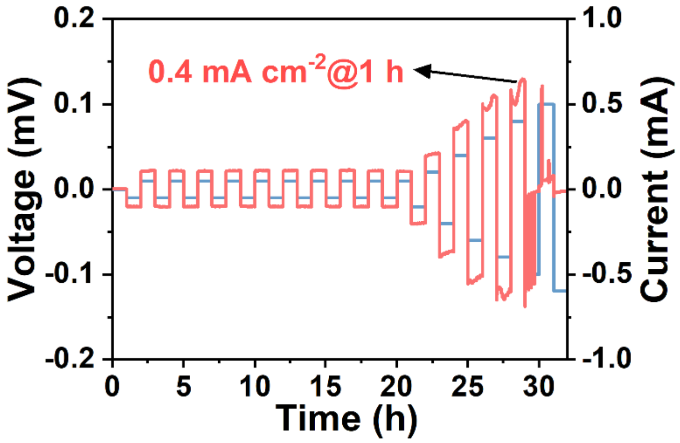

Supplementary Figure 16. CCD measurement of crystalline $Na_5SmSi_4O_{12}$.

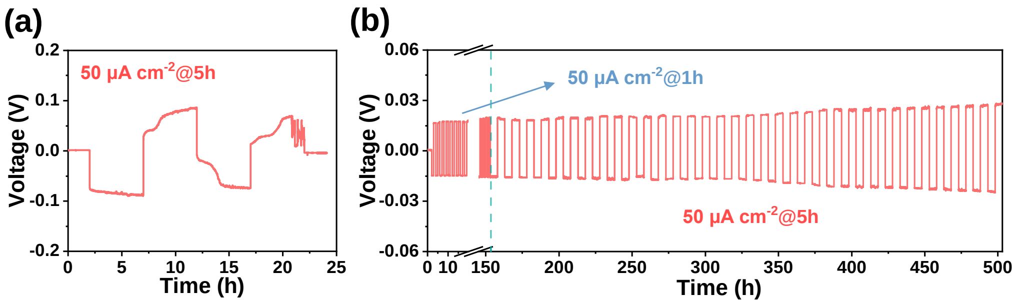

Supplementary Figure 17. Cycling performance of Na|$Na_5SmSi_4O_{12}$|Na at a current density of 0.05 mA cm^{-2} with 10 h per cycle (a) without and (b) with a low area capacity of 0.05 mA h cm^{-2} is applied for 150 h in advance.

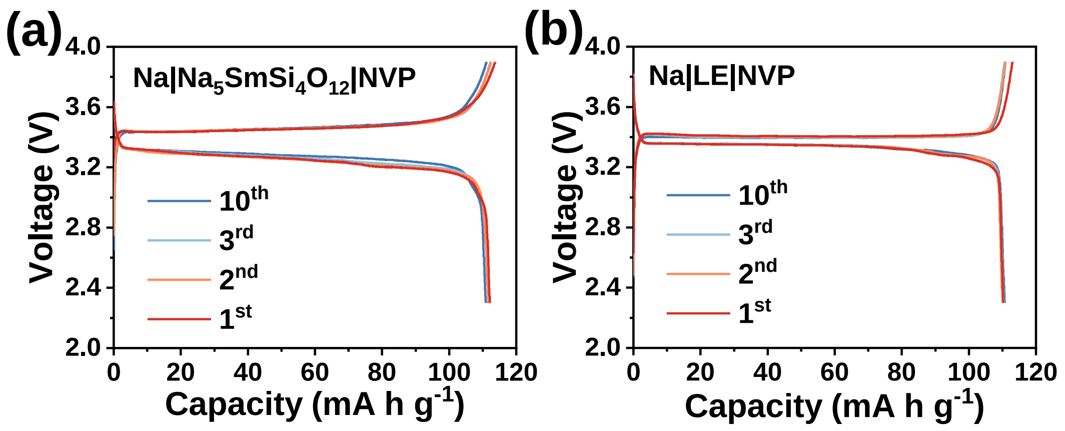

Supplementary Figure 18. The galvanostatic charge-discharge profiles at a current rate of 0.5 C with (a) $Na_5SmSi_4O_{12}$ and (b) liquid electrolyte.

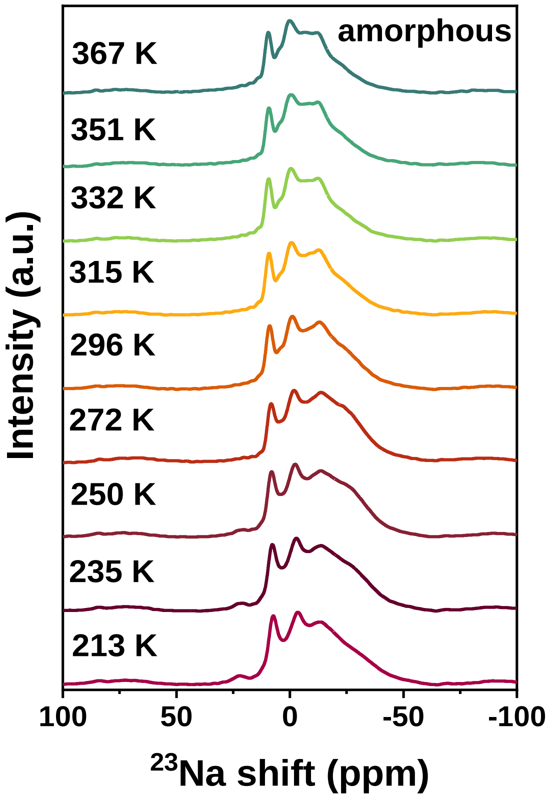

Supplementary Figure 19. Solid-state ^{23}Na NMR spectra of amorphous $Na_5SmSi_4O_{12}$ at different temperatures.

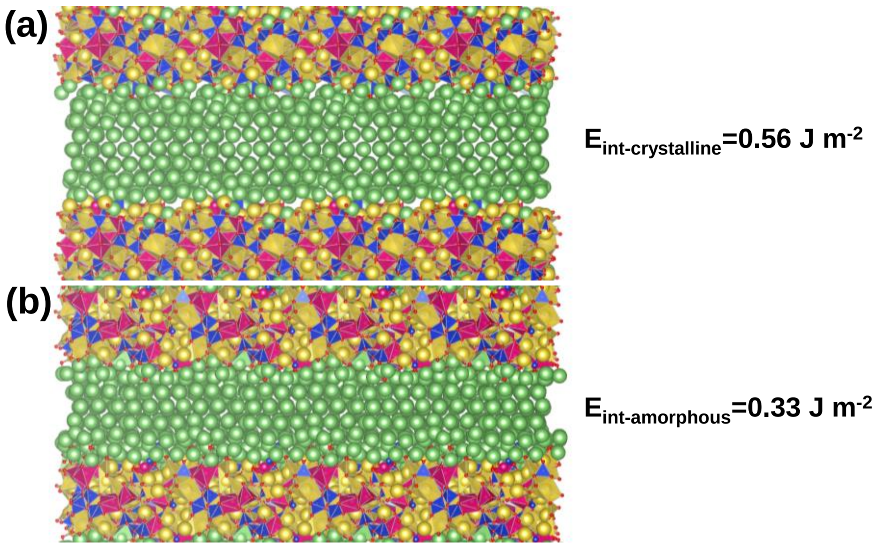

Supplementary Figure 20. Structures of the (a) crystalline $Na_5SmSi_4O_{12}$/Na interface and (b) amorphous $Na_5SmSi_4O_{12}$/Na interface and interfacial energies.

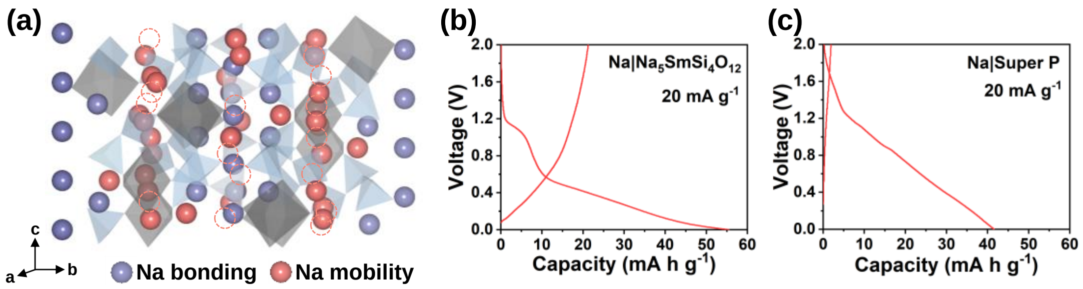

Supplementary Figure 21. (a) Schematical crystal of $Na_5SmSi_4O_{12}$; The initial charge-discharge profile of (b) $Na_5SmSi_4O_{12}$ and (c) super p as the anode in the Na metal half cell.

$Na_5SmSi_4O_{12}$ is investigated as an anode material to validate this possible insertion. The discharge capacity of $Na_5SmSi_4O_{12}$ is demonstrated in Supplementary Figure 21, with the exception of the contribution from Super P, indicating that the SE possesses additional sites capable of accommodating Na^+.

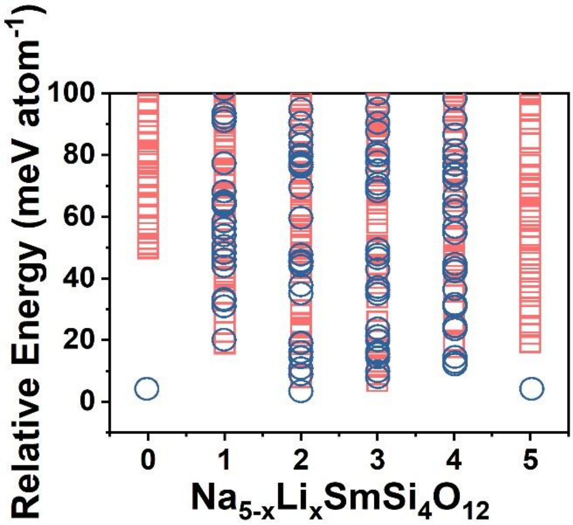

Supplementary Figure 22. Relative energies of $Na_{5-x}Li_xSm_4O_{12}$. All energies are shifted so shat the crystalline end members are at 0 eV.

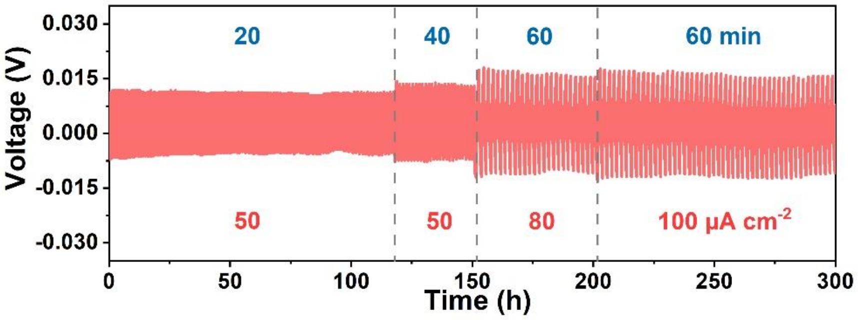

Supplementary Figure 23. Cycling performance of Li|$Na_5SmSi_4O_{12}$|Li at 50 °C with varying current densities.

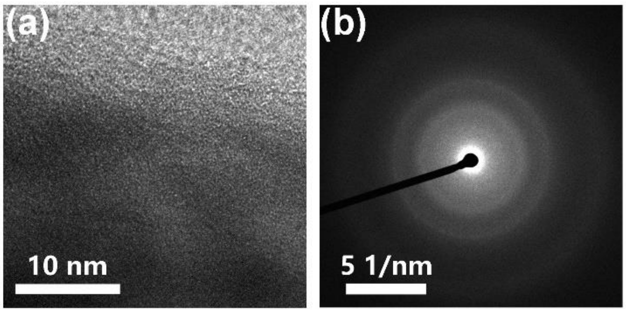

Supplementary Figure 24. (a) HRTEM and (b) SAED patterns of amorphous $Na_{5-x}Li_xSmSi_4O_{12}$.

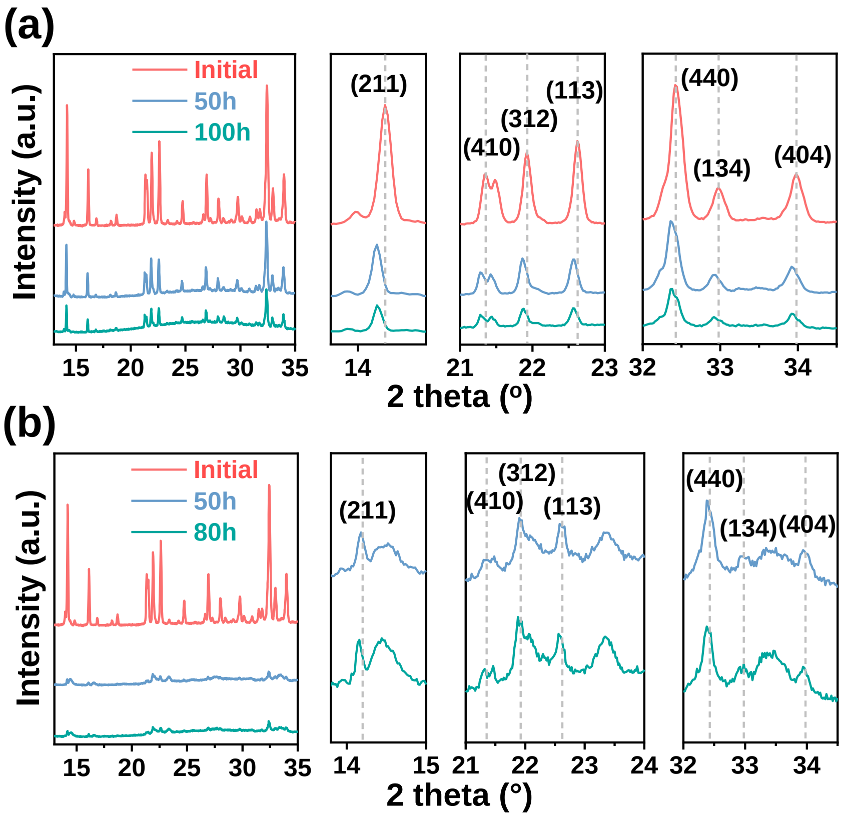

Supplementary Figure 25. XRD profiles of $Na_5SmSi_4O_{12}$ with a slow scanning rate after cycling in (a) Na|$Na_5SmSi_4O_{12}$|Na and (b) Li|$Na_5SmSi_4O_{12}$|Li for different times.

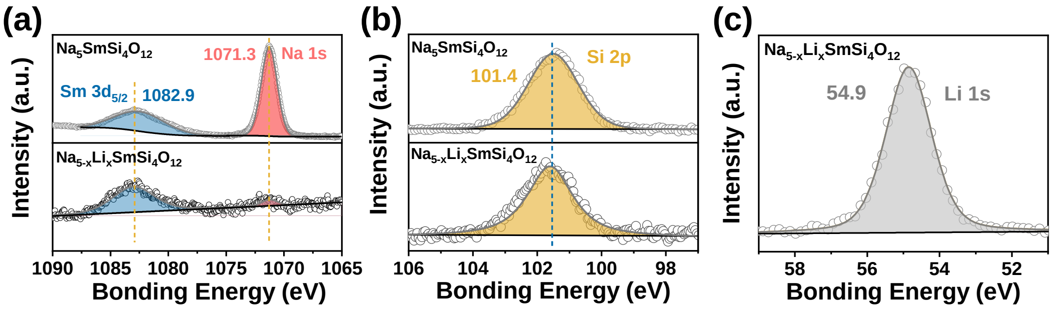

Supplementary Figure 26. (a) Sm 3*d*, Na 1*s*, (b) Si 2*p* and (c) Li 1*s* XPS spectra of $Na_{5-x}Li_xSmSi_4O_{12}$.

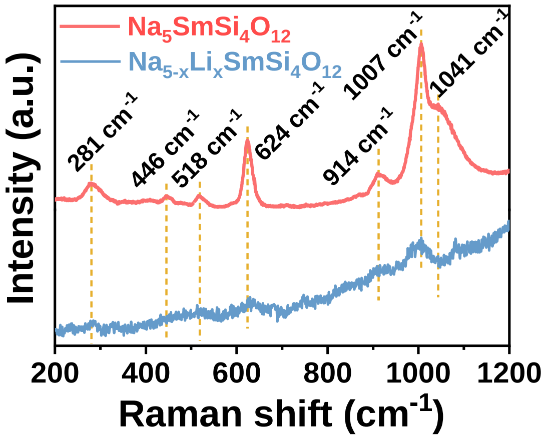

Supplementary Figure 27. Raman spectrum of amorphous $Na_{5-x}Li_xSmSi_4O_{12}$.

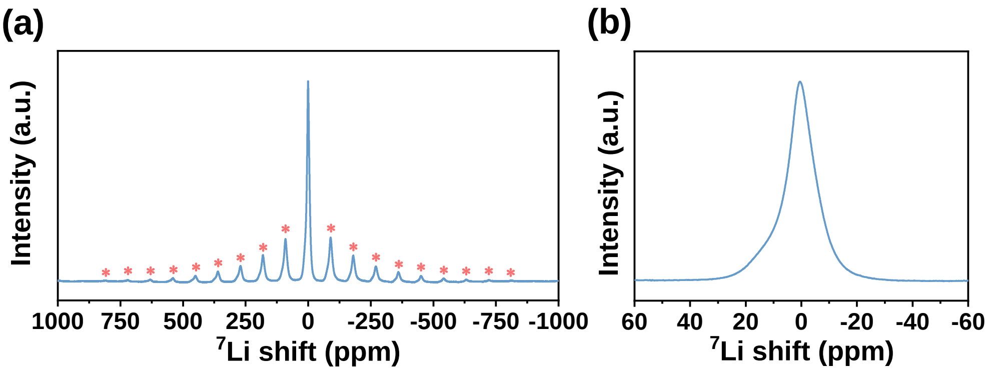

Supplementary Figure 28. Solid-state ^{7}Li NMR spectrum of $Na_{5-x}Li_xSmSi_4O_{12}$ after cycling with Li metal. The full spectrum is shown on the left (a) with many folds of spinning sidebands (SSBs) marked with asterisks. (b) The central isotropic signal enlarged from (a).

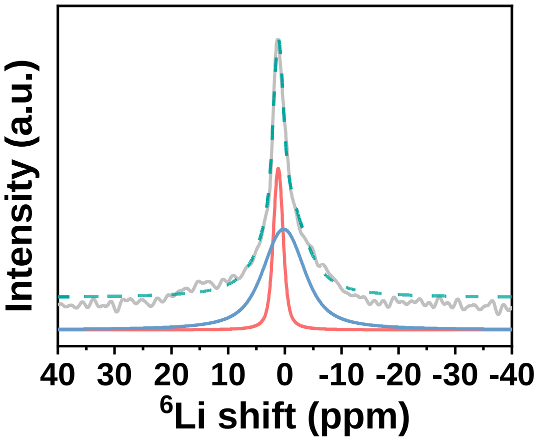

Supplementary Figure 29. Solid-state ^{6}Li NMR spectrum and simulation of the cycled $Na_{5-x}Li_xSmSi_4O_{12}$.

1 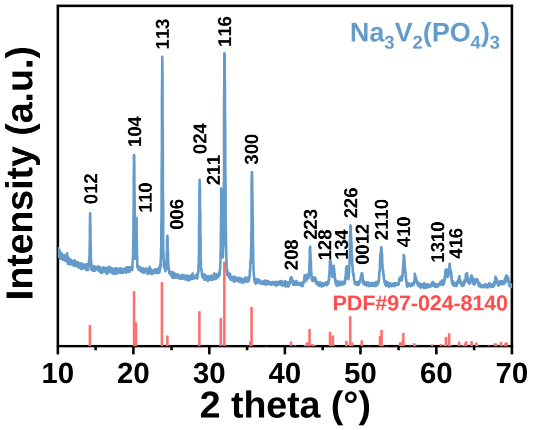

2 **Supplementary Figure 30.** XRD pattern of prepared $Na_3V_2(PO_4)_3$.

Supplementary Table 1. A survey of ionic conductivity and activation energy of oxide electrolytes synthesized with different sintering conditions.

**Solid-state electrolyte**	**Calcination/sintering condition**	**Ionic conductivity at RT (S cm^{-1})**	**Activation energy (eV)**	**Ref.**
$Na_5SmSi_4O_{12}$	800 °C-8 h/950 °C-20 h	2.9×10^{-3} (25°C)	0.15	this work
$Na_{3.4}Zr_{1.9}Zn_{0.1}Si_{2.2}P_{0.8}O_{12}$	900 °C/1250 °C	5.27×10^{-3} (RT)	0.285	1
$Na_5YSi_4O_{12}$	950 °C-8 h/1050 °C-36 h	1.59×10^{-3} (25°C)	0.20	2
$Na_{3.4}Zr_{1.8}Mg_{0.2}PO_{12}$	1000 °C-12 h/1200 °C-12 h	1.05×10^{-3} (RT)	--	3
$Na_3Zr_2Si_2PO_{12}$	950 °C-8 h/1200 °C-12 h	0.60×10^{-3} (25°C)	--	4
$Na_{3.2}Zr_{1.9}Ca_{0.1}Si_2PO_{12}$	600 °C-6 h/950 °C-12 h/ 1250 °C-5 h	1.67×10^{-3} (25°C)	0.29	5
$Na_{3.2}Zr_{1.9}Mg_{0.1}Si_2PO_{12}$	1100 °C-12 h/1105 °C-12 h	8.64×10^{-4} (25°C)	--	6
$Na_3Zr_2Si_2PO_{12}$	1100 °C-9 h/1250 °C-5 h	2.303×10^{-4} (22°C)	0.47	7
$Na_{3.2}Hf_{1.9}Ca_{0.1}Si_2PO_{12}$	900 °C-8 h/1250 °C-16 h	1.07×10^{-3} (RT)	0.199	8
$Na_{3.3}Zr_{1.7}La_{0.3}Si_2PO_{12}$	550 °C-5 h/850 °C-12 h/ 1200 °C-24 h	3.40×10^{-3} (25°C)	0.291	9
$Na_{3.2}Zr_2Si_{2.2}P_{0.8}O_{12}$-0.5NaF	1000 °C-12 h/1150 °C-24 h	3.36×10^{-3} (25°C)	0.25	9
$Na_3Zr_2Si_2PO_{12}$-0.7NaF	1000 °C-12 h/1150 °C-24 h	1.70×10^{-3} (25°C)	0.28	10
$Na_{3.3}Zr_{1.7}La_{0.3}Si_2PO_{12}$	500 °C-2 h/1050 °C-10 h/ 1250 °C-12 h	1.34×10^{-3} (25°C)	--	11
$Na_{3.1}Y_{0.1}Zr_{1.9}Si_2PO_{12}$ -1 wt.% Bi_2O_3	1100 °C-6 h/1100 °C-6 h	1.21×10^{-3} (RT)	--	12
Na-β"-Al_2O_3-1 wt.% ZnO	1100 °C-2 h/1600 °C-0.5 h	5.70×10^{-2} (300 °C)	0.220	13

Supplementary Table 2. Crystallographic data for the prepared $Na_5SmSi_4O_{12}$.

**Crystal System**	Rhombohedral
**Space Group**	R-3c (no.167)
**Lattice Parameter**	a = b = 22.15 Å, c = 12.69 Å
**Volume, Z**	V = 5389.38 Å^3, Z = 6

**atoms**	**wyckoff positions**	**x**	**y**	**z**	**occupancy**
Sm1	18*e*	0	0.7482	0.7500	0.500
Si1	36*f*	0.8999	0.5490	0.3622	1.000
Si2	36*f*	0.5797	0.1600	0.7137	1.000
O1	36*f*	0.9049	0.5899	0.2733	1.000
O2	36*f*	0.0693	0.2024	0.0090	1.000
O3	36*f*	0.2360	0.6176	0.3025	1.000
O4	36*f*	0.8997	0.2612	0.2433	1.000
O5	36*f*	0.8877	0.4630	0.1089	1.000
O6	36*f*	0.8785	0.3667	0.2185	1.000
Na1	36*f*	0.0673	0.1349	0.8637	1.000
Na2	6*b*	0	0	0	0.321
Na3	6*a*	0	0	0.2500	0.189
Na4	18*e*	0.2750	0	0.7500	0.500
Na5	36*f*	0.3227	0.2030	0.2662	0.315
Na6	36*f*	0.3200	0.1436	0.1020	0.324

Anisotropic Betas*10^{-4}

**Atoms**	**B11**	**B22**	**B33**	**B12**	**B13**	**B23**
Sm1	4.3	5.3	44.8	2.1	2.4	1.2
Si1	54.2	33.2	6.6	50.9	-48.6	-15.2
Si2	10.1	28.2	-11.1	14.3	8.8	32.0
O1	-13.3	-10.7	-5.5	-30.5	-105.9	-25.0
O2	64.6	58.3	-3.5	67.5	-10.8	-64.3
O3	27.3	32.8	-78.4	-21.5	-9.1	41.2
O4	43.6	-18.9	-106.2	-43.3	-59.4	12.1

O5	-71.7	51.3	94.2	-47.7	-53.9	-29.0
O6	4.9	38.0	-103.0	-35.2	53.6	99.0
Na1	10.6	12.8	139.2	14.8	71.7	82.8
Na2	32.5	32.5	40.2	16.3	0.0	0.0
Na3	4.0	4.0	119.7	2.0	0.0	0.0
Na4	8.5	81.0	234.4	40.5	67.6	135.2
Na5	-18.2	-10.3	299.8	37.9	-53.0	-102.9
Na6	1.6	-71.7	615.9	17.7	135.6	-59.6

*R_{wp} = 10.7 %, R_p = 8.15 %, R_e = 6.47 %, S = R_{wp} / R_e = 1.65, Chi = 3.59

Supplementary Table 3. Simulations results from ^{23}Na NMR data of $Na_5SmSi_4O_{12}$.

Material	Site	shift (ppm)	C_Q(MHz)	η	proportion	Theoretical proportion
$Na_5SmSi_4O_{12}$	Na5	8.8	0.3	1.0	11%	13.33%
	Na1	4.5	2.4	0.2	43%	40%
	Na3	1.6	0.9	0.4	7%	6.67%
	Na4	-16.8	0.1	0.4	18%	20%
	Na2	-23.4	0.6	1.0	7%	6.67%
	Na6	-28.6	0.8	0.6	14%	13.33%

Supplementary Table 4. Electrolyte bulk, grain boundary and interfacial resistance at 25 °C of Na|$Na_5SmSi_4O_{12}$|Na with different cycling times.

Cycling time	Electrolyte bulk resistance (Ω)	Grain boundary resistance (Ω)	Interfacial resistance (Ω)
0 h	17.2	30.6	2987.0
150 h	18.1	26.7	95.9
200 h	16.0	21.2	47.5
300 h	16.5	21.8	49.2

Supplementary Table 5. The Rietveld refined crystallographic data for the cyclic $Na_5SmSi_4O_{12}$ and CeO_2.

**Current density**	0.05 mA cm^{-2}
**Cycling time**	50 h
**Crystal System**	Rhombohedral
**Space Group**	R-3c (no.167)
**Lattice Parameter**	a = b = 22.17 Å, c = 12.67 Å
**Volume, Z**	V = 5397.25 $Å^3$, Z = 6

**atoms**	**wyckoff positions**	**x**	**y**	**z**	**occupancy**
Sm1	18*e*	0	0.7492	0.7500	0.500
Si1	36*f*	0.8980	0.5458	0.3623	1.000
Si2	36*f*	0.5786	0.1590	0.7109	1.000
O1	36*f*	0.9110	0.5841	0.2885	1.000
O2	36*f*	0.0643	0.1935	0.0032	1.000
O3	36*f*	0.2311	0.6134	0.3048	1.000
O4	36*f*	0.8941	0.2707	0.2233	1.000
O5	36*f*	0.8946	0.4735	0.1280	1.000
O6	36*f*	0.8734	0.3798	0.2260	1.000
Na1	36*f*	0.0738	0.1373	0.8904	1.000
Na2	6*b*	0	0	0	0.321
Na3	6*a*	0	0	0.2500	0.189
Na4	18*e*	0.2737	0	0.7500	0.547
Na5	36*f*	0.3375	0.2103	0.2530	0.488
Na6	36*f*	0.3144	0.1427	0.0549	0.464

*R_{wp} = 11.7 %, R_p = 7.76 %, R_e = 6.20 %, S = R_{wp} / R_e = 1.89, Chi = 3.55

**Current density**			0.05 mA cm^{-2}		
**Cycling time**			100 h		
**Crystal System**			Rhombohedral		
**Space Group**			R-3c (no.167)		
**Lattice Parameter**			a = b = 22.18 Å, c = 12.67 Å		
**Volume, Z**			V = 5399.80 $Å^3$, Z = 6		
**atoms**	**wyckoff positions**	**x**	**y**	**z**	**occupancy**
Sm1	18*e*	0	0.7503	0.7500	0.500
Si1	36*f*	0.8973	0.5450	0.3661	1.000
Si2	36*f*	0.5802	0.1581	0.7083	1.000
O1	36*f*	0.9100	0.5801	0.2869	1.000
O2	36*f*	0.0642	0.1932	0.0192	1.000
O3	36*f*	0.2314	0.6135	0.3149	1.000
O4	36*f*	0.8912	0.2684	0.2332	1.000
O5	36*f*	0.8957	0.4693	0.0917	1.000
O6	36*f*	0.8705	0.3821	0.2241	1.000
Na1	36*f*	0.0730	0.1358	0.8824	1.000
Na2	6*b*	0	0	0	0.321
Na3	6*a*	0	0	0.2500	0.189
Na4	18*e*	0.2736	0	0.7500	0.648
Na5	36*f*	0.3379	0.2101	0.2655	0.569
Na6	36*f*	0.3107	0.1423	0.0654	0.486

*R_{wp} = 9.64 %, R_p = 6.77 %, R_e = 5.85 %, S = R_{wp} / R_e = 1.65, Chi = 2.71

**Current density**	0.05 mA cm^{-2}
**Cycling time**	150 h
**Crystal System**	Rhombohedral
**Space Group**	R-3c (no.167)
**Lattice Parameter**	a = b = 22.18 Å, c = 12.67 Å
**Volume, Z**	V = 5401.94 Å^3, Z = 6

**atoms**	**wyckoff positions**	**x**	**y**	**z**	**occupancy**
Sm1	18*e*	0	0.7502	0.7500	0.500
Si1	36*f*	0.8966	0.5413	0.3749	1.000
Si2	36*f*	0.5811	0.1567	0.7053	1.000
O1	36*f*	0.9097	0.5783	0.2923	1.000
O2	36*f*	0.0602	0.1865	0.0178	1.000
O3	36*f*	0.2414	0.6248	0.3122	1.000
O4	36*f*	0.8988	0.271	0.2175	1.000
O5	36*f*	0.8980	0.4760	0.1401	1.000
O6	36*f*	0.8689	0.3781	0.2370	1.000
Na1	36*f*	0.0711	0.1352	0.8828	1.000
Na2	6*b*	0	0	0	0.321
Na3	6*a*	0	0	0.2500	0.189
Na4	18*e*	0.2788	0	0.7500	0.494
Na5	36*f*	0.3412	0.2182	0.2560	0.575
Na6	36*f*	0.3165	0.1418	0.0769	0.451

*R_{wp} = 11.2 %, R_p = 7.68 %, R_e = 6.55 %, S = R_{wp} / R_e = 1.71, Chi = 2.90

**Current density**	0.05 mA cm^{-2}
**Cycling time**	200 h
**Crystal System**	Rhombohedral
**Space Group**	R-3c (no.167)
**Lattice Parameter**	a = b = 22.18 Å, c = 12.67 Å
**Volume, Z**	V = 5400.83 $Å^3$, Z = 6

**atoms**	**wyckoff positions**	**x**	**y**	**z**	**occupancy**
Sm1	18*e*	0	0.7504	0.7500	0.500
Si1	36*f*	0.8987	0.5415	0.3654	1.000
Si2	36*f*	0.5811	0.1578	0.7111	1.000
O1	36*f*	0.9086	0.5802	0.2900	1.000
O2	36*f*	0.0634	0.1921	0.00625	1.000
O3	36*f*	0.2294	0.6230	0.3002	1.000
O4	36*f*	0.9041	0.2712	0.2303	1.000
O5	36*f*	0.8966	0.4605	0.0994	1.000
O6	36*f*	0.8792	0.3787	0.2238	1.000
Na1	36*f*	0.0695	0.1346	0.8886	1.000
Na2	6*b*	0	0	0	0.321
Na3	6*a*	0	0	0.2500	0.189
Na4	18*e*	0.2776	0	0.7500	0.554
Na5	36*f*	0.3527	0.2190	0.2755	0.490
Na6	36*f*	0.3110	0.1439	0.0513	0.434

*R_{wp} = 7.84 %, R_p = 5.81 %, R_e = 6.09 %, S = R_{wp} / R_e = 1.29, Chi = 1.66

**Current density**	0.1 mA cm^{-2}
**Cycling time**	200 h
**Crystal System**	Rhombohedral
**Space Group**	R-3c (no.167)
**Lattice Parameter**	a = b = 22.17 Å, c = 12.67 Å
**Volume, Z**	V = 5396.56$Å^3$, Z = 6

**atoms**	**wyckoff positions**	**x**	**y**	**z**	**occupancy**
Sm1	18*e*	0	0.74868	0.7500	0.500
Si1	36*f*	0.8925	0.5396	0.3711	1.000
Si2	36*f*	0.5823	0.1581	0.7068	1.000
O1	36*f*	0.9075	0.5725	0.2952	1.000
O2	36*f*	0.0706	0.1938	0.0167	1.000
O3	36*f*	0.2307	0.6116	0.2961	1.000
O4	36*f*	0.8909	0.2656	0.2311	1.000
O5	36*f*	0.8996	0.4846	0.1346	1.000
O6	36*f*	0.8727	0.3771	0.2289	1.000
Na1	36*f*	0.0762	0.1398	0.8867	1.000
Na2	6*b*	0	0	0	0.321
Na3	6*a*	0	0	0.2500	0.189
Na4	18*e*	0.2810	0	0.7500	0.586
Na5	36*f*	0.3368	0.2154	0.2562	0.488
Na6	36*f*	0.3150	0.1387	0.0578	0.494

*R_{wp} = 11.4 %, R_p = 7.33 %, R_e = 6.41 %, S = R_{wp} / R_e = 1.78, Chi = 3.16

**Current density**	0.15 mA cm^{-2}
**Cycling time**	200 h
**Crystal System**	Rhombohedral
**Space Group**	R-3c (no.167)
**Lattice Parameter**	a = b = 22.17 Å, c = 12.68 Å
**Volume, Z**	V = 5396.48 Å^3, Z = 6

**atoms**	**wyckoff positions**	**x**	**y**	**z**	**occupancy**
Sm1	18*e*	0	0.7482	0.7500	0.500
Si1	36*f*	0.8940	0.5395	0.3661	1.000
Si2	36*f*	0.5803	0.1590	0.7098	1.000
O1	36*f*	0.9038	0.5833	0.2946	1.000
O2	36*f*	0.0721	0.1982	0.0115	1.000
O3	36*f*	0.2338	0.6087	0.2947	1.000
O4	36*f*	0.8932	0.2667	0.2345	1.000
O5	36*f*	0.8906	0.4711	0.1235	1.000
O6	36*f*	0.8796	0.3778	0.2244	1.000
Na1	36*f*	0.0727	0.1423	0.8873	1.000
Na2	6*b*	0	0	0	0.321
Na3	6*a*	0	0	0.2500	0.189
Na4	18*e*	0.2837	0	0.7500	0.505
Na5	36*f*	0.3382	0.2143	0.2423	0.459
Na6	36*f*	0.3208	0.1444	0.0476	0.452

*R_{wp} = 6.89 %, R_p = 5.12 %, R_e = 5.65 %, S = R_{wp} / R_e = 1.22, Chi = 1.48

**Crystal System**			Cubic		
**Space Group**			Fm-3m (no.225)		
**Lattice Parameter**			a = b = c = 5.41 Å		
**Volume, Z**			V = 158.41 Å^3, Z = 4		
**atoms**	**wyckoff positions**	**x**	**y**	**z**	**occupancy**
Ce	4a	0	0	0	1
O	8c	0.25	0.25	0.25	1

Supplementary Table 6. A survey of critical current density of Na-based oxide solid-state electrolytes.

Electrolytes	Operating temperature	Critical current density	Deposited capacity	Ref.
**amorphous $Na_5SmSi_4O_{12}$**	**25 °C**	**1.4 mA cm^{-2}**	**1.4 mA h cm^{-2}**	This work
crystalline $Na_5SmSi_4O_{12}$	25 °C	0.4 mA cm^{-2}	0.4 mA h cm^{-2}	
$Na_{4.9}Sm_{0.3}Y_{0.2}Gd_{0.2}La_{0.1}Al_{0.1}Zr_{0.1}Si_4O_{12}$	25 °C	0.6 mA cm^{-2}	0.6 mA cm^{-2}	14
$Na_{3.2}Zr_{1.9}Mg_{0.1}Si_2PO_{12}$	25 °C	0.5 mA cm^{-2}	0.08 mA h cm^{-2}	4
AlF_3-$Na_3Zr_2Si_2PO_{12}$	60 °C	1.2 mA cm^{-2}	2.4 mA h cm^{-2}	5
$Na_3Zr_2Si_2PO_{12}$-10 wt.% $Na_2B_4O_7$	25 °C	0.55 mA cm^{-2}	0.275 mA h cm^{-2}	15
$Na_3Zr_2Si_2PO_{12}$	60 °C	0.4 mA cm^{-2}	0.1 mA h cm^{-2}	16
$Na_{3.4}Mg_{0.1}Zr_{1.9}Si_{2.2}P_{0.8}O_{12}$	60 °C	2.0 mA cm^{-2}	0.5 mA h cm^{-2}	16
$Na_3Zr_2Si_2PO_{12}$	25 °C	0.07 mA cm^{-2}	0.01 mA h cm^{-2}	6
$Na_{3.2}Hf_{1.9}Ca_{0.1}Si_2PO_{12}$@$SnO_2$	60 °C	1.9 mA cm^{-2}	0.475 mA h cm^{-2}	6
$Na_3Zr_2Si_2PO_{12}$	25 °C	0.2 mA cm^{-2}	0.5 mA h cm^{-2}	17
$Na_3Zr_2Si_2PO_{12}$	25 °C	0.4 mA cm^{-2}	0.4 mA h cm^{-2}	18
$Na_{3.2}Hf_{1.9}Ca_{0.1}Si_2PO_{12}$-CuO	25 °C	0.6 mA cm^{-2}	0.6 mA cm^{-2}	18
$Na_3Zr_2Si_2PO_{12}$	25 °C	0.15 mA cm^{-2}	0.15 mA h cm^{-2}	19
$Na_3Zr_2Si_2PO_{12}$	25 °C	0.1 mA cm^{-2}	0.02 mA h cm^{-2}	19
$Na_3Zr_2Si_2PO_{12}$-TiO_2	25 °C	1.0 mA cm^{-2}	0.08 mA h cm^{-2}	20
$Na_3Zr_2Si_2PO_{12}$	25~27 °C	0.6 mA cm^{-2}	0.3 mA h cm^{-2}	21

Supplementary Table 7. A survey of cycle performance of oxide electrolytes-based solid-state cells.

Solid-state cells description	Operating temperature	Cycle performance	Ref.
**Na|$Na_5SmSi_4O_{12}$|$Na_3V_2(PO_4)_3$**	**25 °C**	**4000 cycles 100%**	**This work**
Na|$Na_5YSi_4O_{12}$|$Na_3V_2(PO_4)_3$	25 °C	500 cycles 100%	1
Na|β″-Al_2O_3|$NaTi_2(PO_4)_3$	25 °C	50 cycles 75.1%	22
Na|β″-Al_2O_3|$Na_{0.66}Ni_{0.33}Mn_{0.67}O_2$	70 °C	10000 cycles 90%	23
Na|$Na_3Zr_2(Si_2PO_{12})$|NVP	50 °C	100 cycles 98%	24
Na|$Na_3Zr_2Si_2PO_{12}$|$Na_2MnFe(CN)_6$	60 °C	200 cycles 89.2%	25
Na|beta-alumina|PTO	60 °C	50 cycles 80%	26
Na|$Na_{3.2}Zr_{1.8}Ca_{0.1}Si_2PO_{12}$|NVP	25 °C	500 cycles 98%	3
Na|$Na_{3.4}Zr_{1.8}Mg_{0.2}PO_{12}$|$NaCrO_2$	25 °C	1755 cycles 87%	7
Na|polydopamine-$Na_{3.4}Zr_{1.9}Zn_{0.1}Si_{2.2}P_{0.8}O_{12}$|$FeS_2$	60 °C	300 cycles 73.3%	12
UW-Na|$Na_3Zr_2Si_2PO_{12}$|NVP	25−27 °C	900 cycles 89.8%	21
Na|$Na_{3.3}Zr_{1.7}La_{0.3}Si_2PO_{12}$|IL|NVP	25 °C	10000 cycles 100%	8
Na|$Na_3Zr_2Si_2PO_{12}$|NVP	25 °C	100 cycles 97%	19

Supplementary Table 8. The comparison of element ratios of $Na_5SmSi_4O_{12}$ before and after cycling.

	**Na**	**Sm**	**Si**	**O**
**Before cycling**	4.9	1.0	4.2	12.1
**After cycling**	6.1	1.0	4.1	12.2

References

1. Sun G, *et al.* $Na_5YSi_4O_{12}$: A sodium superionic conductor for ultrastable quasi-solid-state sodium-ion batteries. *Energy Storage Mater.* **41**, 196-202 (2021).
2. Matios E, *et al.* Graphene Regulated Ceramic Electrolyte for Solid-State Sodium Metal Battery with Superior Electrochemical Stability. *ACS Appl. Mater. Interfaces* **11**, 5064-5072 (2019).
3. Lu Y, Alonso JA, Yi Q, Lu L, Wang ZL, Sun C. A High-Performance Monolithic Solid-State Sodium Battery with Ca^{2+} Doped $Na_3Zr_2Si_2PO_{12}$ Electrolyte. *Adv. Energy Mater.* **9**, 1901205 (2019).
4. Fu H, *et al.* Reducing Interfacial Resistance by Na-SiO_2 Composite Anode for NASICON-Based Solid-State Sodium Battery. *ACS Mater. Lett.* **2**, 127-132 (2019).
5. Miao X, *et al.* AlF_3-modified anode-electrolyte interface for effective Na dendrites restriction in NASICON-based solid-state electrolyte. *Energy Storage Mater.* **30**, 170-178 (2020).
6. Tian H, Liu S, Deng L, Wang L, Dai L. New-type Hf-based NASICON electrolyte for solid-state Na-ion batteries with superior long-cycling stability and rate capability. *Energy Storage Mater.* **39**, 232-238 (2021).
7. Wang C, *et al.* Grain Boundary Design of Solid Electrolyte Actualizing Stable All-Solid-State Sodium Batteries. *Small* **17**, e2103819 (2021).
8. Zhang Z, *et al.* A Self-Forming Composite Electrolyte for Solid-State Sodium Battery with Ultralong Cycle Life. *Adv. Energy Mater.* **7**, 1601196 (2017).
9. Shao Y, *et al.* A novel NASICON-based glass-ceramic composite electrolyte with enhanced Na-ion conductivity. *Energy Storage Mater.* **23**, 514-521 (2019).
10. Sun F, *et al.* Insight into Ion Diffusion Dynamics/Mechanisms and Electronic

Structure of Highly Conductive Sodium-Rich $Na_{3+x}La_xZr_{2-x}Si_2PO_{12}$ ($0 \leq x \leq 0.5$) Solid-State Electrolytes. *ACS Appl. Mater. Interfaces* **13**, 13132-13138 (2021).

11. Miao RJ, Cao XG, Wang WG, Zhang HY. Influence of Bi_2O_3 additive on the electrochemical performance of $Na_{3.1}Y_{0.1}Zr_{1.9}Si_2PO_{12}$ inorganic solid electrolyte. *Ceram. Int.* **47**, 17455-17462 (2021).
12. Yang J*, et al.* Ultrastable All-Solid-State Sodium Rechargeable Batteries. *ACS Energy Lett.* **5**, 2835-2841 (2020).
13. Zhang T, Wang Z, Feng X, Xie Z, Li Y. Preparation and characterization of ZnO-doped and Li_2O-stabilized Na-β"-Al_2O_3 solid electrolyte via a solid-state reaction method. *J. Mater. Sci.: Mater. Electron.* **32**, 14149-14155 (2021).
14. Sun G*, et al.* High-Entropy Solid-State Na-Ion Conductor for Stable Sodium-Metal Batteries. *Chem. Eur. J.* **29**, e202300413 (2023).
15. Zhao Y, Wang C, Dai Y, Jin H. Homogeneous Na^+ transfer dynamic at Na/$Na_3Zr_2Si_2PO_{12}$ interface for all solid-state sodium metal batteries. *Nano Energy* **88**, 106293 (2021).
16. Shen L, Yang J, Liu G, Avdeev M, Yao X. High ionic conductivity and dendrite-resistant NASICON solid electrolyte for all-solid-state sodium batteries. *Mater. Today Energy* **20**, 100691 (2021).
17. Wang C, Jin H, Zhao Y. Surface Potential Regulation Realizing Stable Sodium/$Na_3Zr_2Si_2PO_{12}$ Interface for Room-Temperature Sodium Metal Batteries. *Small* **17**, e2100974 (2021).
18. Sun Z*, et al.* Active Control of Interface Dynamics in NASICON-Based Rechargeable Solid-State Sodium Batteries. *Nano Lett.* **22**, 7187-7194 (2022).
19. Yang J*, et al.* Improving Na/$Na_3Zr_2Si_2PO_{12}$ Interface via SnO_x/Sn Film for High-Performance Solid-State Sodium Metal Batteries. *Small Methods* **5**,

e2100339 (2021).

20. Gao Z*, et al.* TiO_2 as Second Phase in $Na_3Zr_2Si_2PO_{12}$ to Suppress Dendrite Growth in Sodium Metal Solid-State Batteries. *Adv. Energy Mater.* **12**, 2103607 (2022).

21. Wang X, Chen J, Wang D, Mao Z. Improving the alkali metal electrode/inorganic solid electrolyte contact via room-temperature ultrasound solid welding. *Nat. Commun.* **12**, 7109 (2021).

22. Zhao K*, et al.* A room temperature solid-state rechargeable sodium ion cell based on a ceramic Na-β″-Al_2O_3 electrolyte and $NaTi_2(PO_4)_3$ cathode. *Electrochem. Commun.* **69**, 59-63 (2016).

23. Liu L*, et al.* Toothpaste-like Electrode: A Novel Approach to Optimize the Interface for Solid-State Sodium-Ion Batteries with Ultralong Cycle Life. *ACS Appl. Mater. Interfaces* **8**, 32631-32636 (2016).

24. Gao H, Xue L, Xin S, Park K, Goodenough JB. A Plastic-Crystal Electrolyte Interphase for All-Solid-State Sodium Batteries. *Angew. Chem. Int. Ed. Engl.* **56**, 5541-5545 (2017).

25. Gao H, Xin S, Xue L, Goodenough JB. Stabilizing a High-Energy-Density Rechargeable Sodium Battery with a Solid Electrolyte. *Chem* **4**, 833-844 (2018).

26. Chi X*, et al.* A high-energy quinone-based all-solid-state sodium metal battery. *Nano Energy* **62**, 718-724 (2019).
